# The Effect of Aloe Vera, Pomegranate Peel, Grape Seed Extract, Green Tea, and Sodium Ascorbate as Antioxidants on the Shear Bond Strength of Composite Resin to Home-bleached Enamel

**Published:** 2015-12

**Authors:** Farahnaz Sharafeddin, Farnaz Farshad

**Affiliations:** a Biomaterial Research Center, Dept. of Operative Dentistry, School of Dentistry, Shiraz University of Medical Sciences, Shiraz, Iran.; b Postgraduate Student, Student Research Committee, Dept. of Operative Dentistry, School of Dentistry, Shiraz University of Medical Sciences, Shiraz, Iran.

**Keywords:** Antioxidant, Shear Bond Strength, Tooth Bleaching, Composite Restoration

## Abstract

**Statement of the Problem:**

Immediate application of bonding agent to home- bleached enamel leads to significant reduction in the shear bond strength of composite resin due to the residual oxygen. Different antioxidant agents may overcome this problem.

**Purpose:**

This study aimed to assess the effect of different antioxidants on the shear bond strength of composite resin to home-bleached.

**Materials and Method:**

Sixty extracted intact human incisors were embedded in cylindrical acrylic resin blocks (2.5×1.5 cm), with the coronal portion left out of the block. After bleaching the labial enamel surface with 15% carbamide peroxide, they were randomly divided into 6 groups (n=10). Before performing composite resin restoration by using a cylindrical Teflon mold (5×2 mm), each group was treated with one of the following antioxidants: 10% sodium ascorbate solution, 10% pomegranate peel solution, 10% grape seed extract, 5% green tea extract, and aloe vera leaf gel. One group was left untreated as the control. The shear bond strength of samples was tested under a universal testing machine (ZwickRoell Z020). The shear bond strength data were analyzed by one-way ANOVA and post hoc Tukey tests (*p*< 0.05).

**Results:**

No significant difference existed between the control and experimental groups. Moreover, there was no statistically significant difference between the effects of different antioxidants on the shear bond strength of bleached enamel.

**Conclusion:**

Different antioxidants used in this study had the same effect on the shear bond strength of home-bleached enamel, and none of them caused a statistically significant increase in its value.

## Introduction

Vital tooth bleaching generally involves application of hydrogen peroxide on the tooth surface in an office technique or application of carbamide peroxide in home technique.[1-2]

Carbamide peroxide and hydrogen peroxide function as oxidative agents by forming free radicals, oxygen reactive molecules, and hydrogen ions. These active molecules attack the pigments that are present in the teeth and remove them; the reason we can observe their effectiveness in whitening of the teeth.[3-5]

In home bleaching technique, carbamide peroxide is used for 0.5‒8h/day depending on its concentration under a dentist’s supervision.[3-5]

Bleaching treatment leaves some side effects on the teeth namely decreasing the bonding ability, causing morphological changes in enamel and dentin surface, reducing enamel wear resistance, and causing surface roughness. It also increases enamel porosity and changes the enamel and dentin mechanical features such as fracture toughness which may reduce the tooth crack resistance and strength.[6]

The shear bond strength (SBS) of composite resin bonded to the tooth surface decreases dramatically right after bleaching treatment. This reduction in SBS is related to the residual peroxides, the presence of which interfere with resin tag formation and the resin bond to the tooth, and subsequently impede the polymerization of resin monomers. The rest of oxygen disperse gradually, and after an appropriate time period (24 hours to 4 weeks), the composite resin recover its bond strength.[7-9]

There are some techniques to prevent the reduction of composite resins bond strength after bleaching, such as removing superficial enamel and application of adhesives which contain organic solutions, alcohol, or antioxidants.[2, 8-9]

Application of antioxidants was reported to be a reversal in the decline of SBS of composite resin to bleached enamel due to its protective role against free radical reactions.[10] Sodium ascorbate, grape seed extract, green tea, pomegranate peel extract, and aloe vera are some antioxidants.[8-9,11-13]

Sodium ascorbate is a neutral, nontoxic and biocompatible antioxidant that improves the bond strength of bleached enamel.[10] The grape seed extract contains oligomeric proanthocyanidin complex which is more potent than sodium ascorbate. Antioxidant activity of dry green tea leaves is related to flanavols.[6, 14-15]

Pomegranate peel extract contains effective compounds such as polyphenols whose antioxidant benefits preponderate over green tea. Likewise, the antioxidant effect of aloe vera is attributed to the polysaccharides found in the leaf gel.[7, 11-13]

Sasaki *et al.* showed that 10% sodium ascorbate could not reverse the oxidizing effect of 10% carbamide peroxide on enamel and could not increase the SBS.[10] However, in another study, carbamide peroxide bleaching was followed by application of sodium ascorbate hydrogel and it was found to have increased the SBS of bleached enamel, which was proportional to the duration of application.[16]

No published study has compared the effectiveness of different antioxidants on the SBS of home-bleached enamel. The aim of this in-vitro study was to evaluate and contrast the effects of different antioxidants, including aloe vera, green tea, pomegranate peel extract, grape seed extract, and sodium ascorbate on the SBS of home-bleached enamel. 

## Materials and Method

In this experimental study, 60 recently extracted sound human maxillary incisors were collected and randomly divided into 6 groups (n=10). The tooth roots were embedded in cylindrical acrylic resin blocks (1.5×2.5 cm), with only the tooth coronal portion (above the cementoenamel junction) out of the block. The labial enamel surfaces of samples (6×6 mm) were polished with silicon carbide paper of 600 grit size (Moyco Precision Abrasives; Montgomeryville, PA, USA). The prepared labial surfaces of all the teeth were bleached with 15% carbamide peroxide gel (Opalescence; 15% PF, Ultradent Product Inc, South Jordan, UT, USA) for 6h/day for 5 days. After completion of daily bleaching procedures, the teeth were rinsed with water spray for 1 minute and then kept in distilled water at room temperature until the next day.

**Figure 1:**
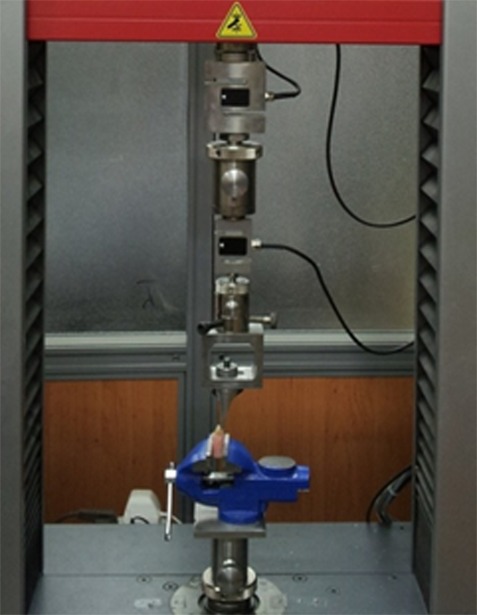
The samples under the SBS test in the universal testing machine

By solving a 10-mg sodium ascorbate pill (Vitamin C; DR Muller, Germany), a pomegranate peel extract pill (Amin Pharma; Iran), a grape seed extract pill (Enerex; Canada) and a 5-mg green tea extract pill (CAMGREEN; Giah Essence, Iran) in 100 mL of distilled water, 4 solutions were prepared. The resultant solutions were 10% sodium ascorbate solution, 10% pomegranate peel extract solution, 10% grape seed extract solution, and 5% green tea solution, respectively. Aloe vera gel was prepared from the aloe vera inner leaf. The study groups were named alphabetically from A to F. In group A (control group), immediately after bleaching, composite resin restorative procedure was carried out. In groups B, C, D, and E immediately after bleaching, a solution of 10% sodium ascorbate, 10% pomegranate peel, 10% grape seed extract, 5% green tea, and aloe vera leaf gel were respectively applied on the bleached enamel surface for 10 minutes. Then the samples were rinsed with water for 30 seconds and dried. Finally, composite resin build-up procedure was done. All the bleached surfaces were etched by using 37% phosphoric acid gel for 15 seconds (Total Etching Gel; Ivoclar Vivadent, Schaan, Liechtenstein). Then they were rinsed with water spray for 15 seconds, Adper Single Bond (3M ESPE; Dental Products, St Paul, MN, USA) was applied, and light-cured by an LED unit (Demi Plus; Kerr, Switzerland) for 20 seconds at a light intensity of 1200 W/cm^2^. Finally, the restoration procedure was done with composite resin (Filtek Z350; 3M Dental Products) by using a Teflon mold (5×2 mm). They were light-cured for 20 seconds by the same LED light-curing unit. All the samples were immersed in distilled water at room temperature for 24 hours. For measuring the shear bond strength, the samples were placed in a universal testing machine (ZwickRoell Testing Machine Z020; Germany) with a blade-shaped tip, and the force was applied to the composite resin‒enamel interface at a crosshead speed of 0.5 mm/min (Figure 1).

Data were analyzed by using one-way ANOVA and post-hoc Tukey’s tests (*p*< 0.05).

## Results

The values of Mean±SD shear bond strength of all the 6 groups are represented in Table 1.

**Table 1:** Descriptive statistics of the shear bond strength (MPa) obtained from the study groups

** Groups**	** Mean (SD)**
A (Control)	12.14 (2.30)
B (Sodium Ascorbate)	13.37 (1.68)
C (Pomegranate Peel)	13.49 (1.29)
D (Grape Seed Extract)	13.49 (1.88)
E (Green Tea)	13.76 (0.81)
F (Aloe Vera)	13.48 (1.65)

**Figure 2:**
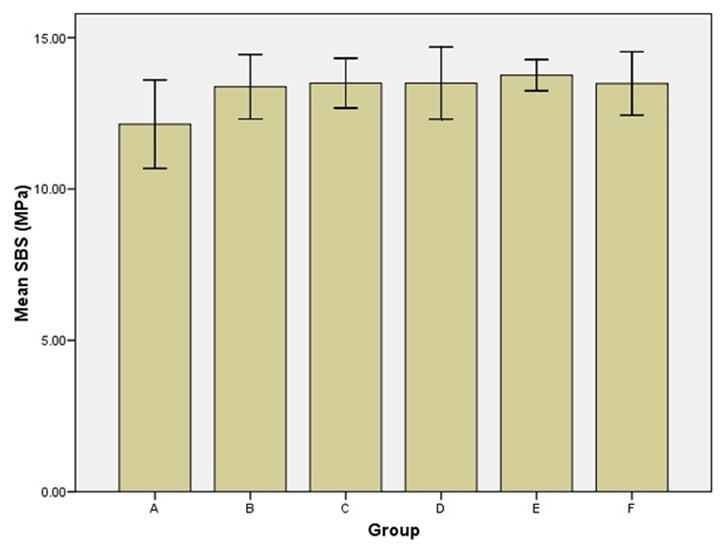
The shear bond strength of the study groups. Groups: A (Control), B (Sodium ascorbate), C (Pomegranate peel), D (Grape seed extract), E (Green tea), F (Aloe vera).

ANOVA was used to analyze the differences between the mean SBS of study groups, and Tukey’s post-hoc test was used to determine the significant differences between the mean values of the groups. One-way ANOVA revealed no statistically significant difference between the control group and groups treated with antioxidants (*p*< 0.05). Similar mean values were obtained from the groups treated by different antioxidants (Figure 2).

## Discussion

The decrease in the shear bond strength of a bleached tooth is attributed to the residual peroxides which interfere with resin tag formation and the adhesion of resin to the tooth, and consequently inhibit the polymerization of resin monomers.[5, 17]

Dabas *et al.* performed a study to evaluate the effects of sodium ascorbate concentration and application time on the bond strength of composite resin to bleached enamel. The results revealed that using 10% and 20% sodium ascorbate hydrogel for 30, 60, and 120 minutes, after bleaching with 17% carbamide peroxide, increased the bond strength of composite resin-enamel. The above-mentioned effects were directly related to the application time; however, there was no relation with the concentration.[5]

In the present study, antioxidant solutions were used at 5% and 10% concentrations for 10 minutes and no significant difference was observed between the SBS of control group and the groups treated with antioxidants. While, in their study, 10% and 20% hydrogel was used for 30, 60, and 120 minutes, which might explain the differences in the results. In our study, 15% carbamide peroxide was used as the bleaching agent, whereas, Dabas *et al.* employed 17% carbamide peroxide, which can produce more peroxide molecules.

Danesh Sani and Esmaili investigated the effect of 10% sodium ascorbate hydrogel and the influence of delaying the bonding procedure on the SBS of composite resin and SBS of resin-modified glass-ionomer (RMGI). The enamel was bleached with 9.5% hydrogen peroxide for 6h/day for 7 consecutive days. They found that the SBS decreased when the samples were restored with composite resin immediately after bleaching; in addition, RMGI did not bond to the bleached enamel immediately after bleaching. Therefore, application of sodium ascorbate increased the SBS of composite resin restorations to the enamel bleached with 9.5% hydrogen peroxide.[17] In the current study, 15% carbamide peroxide was used 6h/day for 5 consecutive days as the bleaching agent; while, in the above-mentioned study, hydrogen peroxide was used which is more potent than carbamide peroxide. It can produce more residual oxygen molecules that would significantly decrease the SBS. This might explain the significant effect of antioxidant treatment in that study.

One study showed the positive effects of 10% sodium ascorbate gel on the bonding capacity to enamel bleached with 10% carbamide peroxide. Applying 10% sodium ascorbate for 10, 60, 120, 240, and 480 minutes on bleached enamel, they reported that increasing the application time of the antioxidant improved the SBS of composite resin to enamel. For maximum effectiveness, the antioxidant gel needed to be applied to enamel for at least 60 minutes. In the group on which the antioxidant was practiced for 10 minutes, no significant increase was observed in the SBS; which is consistent with the results of the present study.[8] In our study, 15% carbamide peroxide was used, which is more potent than 10% carbamide peroxide, and the antioxidant was applied for 10 minutes. Accordingly, it seems that by increasing the concentration of bleaching agent, the application time of antioxidant should be increased.

Researchers investigated the effects of 10% sodium ascorbate as an antioxidant on the SBS of composite resin to enamel bleached with either 35% hydrogen peroxide or 16% carbamide peroxide. It was detected that application of 10% sodium ascorbate for 1 minute increased the SBS.[18-19] In the present study, 15% carbamide peroxide and 10% sodium ascorbate were used for 10 minutes; whereas, the bleaching agents used in the above-mentioned study were more potent and could produce more residual oxygen molecules. That was the reason the antioxidant agent had a positive effect in that study.

It was reported that application of only 10% sodium ascorbate raised the SBS of dual-cured resin cement to enamel bleached with carbamide peroxide, and that delaying the bonding procedure had no effect on increasing the SBS.[20] On the other hand, another study reported that application of 10% sodium ascorbate and delaying the bonding process increased the bond strength.[9, 21]

Vidhya *et al.* assessed the neutralizing effect of 5% grape seed solution on the bond strength of enamel bleached with 38% hydrogen peroxide. They concluded that using this solution for 10 minutes could completely neutralize the effect of bleaching agents. In the present study, 15% carbamide peroxide and 5% grape seed solutions were used for 10 minutes but they used 38% hydrogen peroxide in their study. This agent is more potent and can produce more residual oxygen molecules, resulting in a significant decrease in SBS and subsequently the reason for the significant effect of antioxidant treatment in their study.[6]

Another study showed that 6.5% grape seed solution improved the SBS of enamel bleached with 35% carbamide peroxide. It is known that 35% carbamide peroxide produces more residual oxygen molecules, the reason that in their study the neutralizing effect of grape seed extract was more obvious.[22]

Arumugam *et al.* reported that application of 600, 800 and 1000 μmol of green tea solution for 10 and 20 minutes did not increase the SBS of composite resin to enamel bleached with 30% hydrogen peroxide.[23] In our study, although 15% carbamide peroxide was used and 5% green tea solution was applied for 10 minutes, similar results were achieved. Maybe we should have tried different concentrations and longer application times or pure antioxidants to achieve optimal results.

Another study showed that application of pomegranate peel extract, grape seed extract, green tea, and sodium ascorbate on enamel bleached with 40% hydrogen peroxide neutralized the effect of residual oxygen molecules on the bleached enamel surface, and increased the SBS of composite resin.[24] The concentration of antioxidants were similar to the current study, but we used 15% carbamide peroxide as the bleaching agent which is weaker than hydrogen peroxide and could produce less residual oxygen molecules. Apparently, the effect of antioxidant on the SBS would decrease as the bleaching agent concentration decreases.

The statistical analysis in our study revealed that antioxidant treatment of carbamide peroxide-bleached enamel did not affect the SBS of composite resin restoration and treatment with different antioxidants yielded the same results. Further studies should be conducted to evaluate the effect of different concentrations, application times, and type of antioxidants on SBS of bleached enamel. 

## Conclusion

Under the limitation of this *in-vitro* study, it can be concluded that aloe vera, pomegranate peel, grape seed extract, green tea, and sodium ascorbate treatments had no significant effects on the SBS of composite resin to enamel bleached with carbamide peroxide. Moreover, different antioxidants in this study exerted the same effects on the shear bond strength of home-bleached enamel.
